# A Hitchhiker's Guide to Worldwide COVID-19 Vaccinations: A Detailed Review of Monovalent and Bivalent Vaccine Schedules, COVID-19 Vaccine Side Effects, and Effectiveness Against Omicron and Delta Variants

**DOI:** 10.7759/cureus.29837

**Published:** 2022-10-02

**Authors:** Lokesh Goyal, Miana Zapata, Kunal Ajmera, Prabal Chourasia, Ramesh Pandit, Trupti Pandit

**Affiliations:** 1 Hospital Medicine, CHRISTUS Spohn Hospital Corpus Christi - Shoreline, Corpus Christi, USA; 2 Internal Medicine, University of the Incarnate Word School of Osteopathic Medicine, Corpus Christi, USA; 3 Hospital Medicine, Sentara Northern Virginia Medical Center, Woodbridge, USA; 4 Department of Hospital Medicine, Mary Washington Hospital, Fredericksburg, USA; 5 Medicine, Independent Researcher, Philadelphia, USA; 6 Hospital Medicine, University of Pennsylvania/Chester County Hospital, Philadelphia, USA; 7 Pediatrics, Nemours Children's Health, Glen Mills, USA

**Keywords:** bivalent covid-19 vaccine booster, wiv04 and hb02, gam-covid-vac/sputnik v, ad5-based covid-19 vaccine, astrazeneca, moderna, pfizer, delta variant, omicron variant, covid-19 vaccination

## Abstract

For the primary prevention of coronavirus disease 2019 (COVID-19), there are currently four different vaccines available in the USA. These are Pfizer (messenger RNA [mRNA]), Moderna (mRNA), Novavax (recombinant protein), and Jansen/Johnson & Johnson (adenoviral vector). All individuals should get vaccinated, and the Centers for Disease Control and Prevention (CDC) has provided comprehensive guidelines on recommended doses, their frequency by age group, and vaccine types, all discussed in detail in this article. Vaccines are a critical and cost-effective tool for preventing the disease. Prior to receiving a vaccine, patients should get adequate counseling regarding any potential adverse effects post vaccination. Appropriate safety precautions must be taken for those more likely to experience adverse consequences. Healthcare professionals should be aware of the symptoms, indicators, and treatment of any adverse event post-vaccination. We have provided a comprehensive review of the different characteristics of COVID-19 vaccines available in the United States, including their effectiveness against various variants, adverse effects, and precautions necessary for healthcare professionals and the general population. This article also briefly covers COVID-19 vaccines available worldwide, specifically their mode of action and effectiveness.

## Introduction and background

After being discovered in Wuhan, China, in November 2019, the novel coronavirus pandemic expanded quickly, causing significant morbidity, mortality, and financial burden worldwide [1]. Its symptoms range from asymptomatic infection to a life-threatening condition complicated by severe acute respiratory distress syndrome, thromboembolism, and multiorgan failure [2]. As severe acute respiratory syndrome coronavirus 2 (SARS-CoV-2) variants have emerged, our comprehension of coronavirus disease 2019 (COVID-19) infection and management has continued to advance. An effective component of management that has dramatically helped reduce COVID-19 mortality and morbidity has been the introduction of COVID-19 vaccines [3].

The Food and Drug Administration (FDA) first issued emergency use authorization of the COVID-19 vaccine on December 11, 2020, in the United States. Since then, the number of COVID-19 vaccines approved for use has significantly increased worldwide. Along with the development of multiple COVID-19 vaccines, concerns about their side effects and contraindications have also increased. The development of COVID-19 vaccines is regulated by data safety and monitoring committees (DSMCs) composed of vaccine specialists and study sponsors. Before sanctioning the progression of a specific vaccine to the next phase, these groups evaluate the adverse events identified in each clinical study. This review article aims to provide a thorough analysis of COVID-19 vaccines approved for use in the USA, their characteristics, and their adverse effects profile.

## Review

Methodology

A complete review of the medical literature was performed systematically with the keywords "COVID-19," "COVID-19 variants", "COVID-19 vaccine", and "COVID vaccine adverse effects" in PubMed, Google Scholar, and ScienceDirect databases. We also reviewed information from regulatory drug agencies and pharmaceutical companies' websites till September 4, 2022. To gather relevant content, all articles, which included literature reviews, meta-analyses, case reports, case-control cohort studies, and randomized controlled trials of vaccines, were screened and used to complete this article. Data from the Centers for Disease Control and Prevention (CDC) was also used and included in this article. We summarized and organized pertinent information for ease of understanding.

Discussion

General Principles for COVID-19 Vaccine

The COVID-19 virus and its variants have surface spike protein (S-protein) which bind to the angiotensin-converting enzyme 2 (ACE2) receptors on the target cells resulting in membrane fusion. This enables the virus to penetrate the host cell and begin to replicate. The S-protein is the primary antigenic target site of COVID-19 vaccines. The antibodies produced by COVID-19 vaccines bind to the receptor binding domain of the S-protein, preventing the virus from binding to the host [4]. Based on these principles, COVID-19 vaccines evoke a substantial response against subsequent COVID-19 virus infection and offer protection by reducing the risk of severe illness, hospitalizations, and death [5,6]. COVID-19 vaccines, similar to most respiratory virus vaccines, are administered intramuscularly (IM) to evoke a strong systemic immune response [7,8]. COVID-19 vaccines that can be delivered intranasally or respiratory tract are currently under development [9].

COVID-19 Vaccines Available in the United States

Presently, four COVID-19 vaccines are approved for use in the United States by the FDA. They include two mRNA vaccines, an adjuvant recombinant vaccine and an adenoviral vaccine as seen in Table 1.

**Table 1:** Types of COVID-19 vaccine available in the United States. Four different COVID-19 vaccines available in the United States [10-13].

Vaccine Name	Vaccine Type	Age Eligibility
Pfizer-BioNTech (BNT162b2): Comirnaty	mRNA - Bivalent	6 months and older
Moderna (mRNA-1273): Spikevax	mRNA	6 months and older
Novavax (NVX-CoV2373): Nuvaxovid	adjuvanted recombinant protein	18 years or older
Janssen or Johnson & Johnson (Ad26.COV2.S)	Non-replicating viral vector	18 years or older

Why vaccinate? Who to vaccinate? Which vaccine to choose? 

The COVID-19 vaccines effectively reduce individuals' risk of contracting the virus. In addition, research has established that individuals receiving COVID-19 vaccines have a reduced risk of COVID-19-associated morbidity and mortality [3,14]. 

Based on currently available data and CDC guidelines, COVID-19 vaccinations are recommended for all people aged six months and older [15,16]. mRNA COVID-19 vaccines, i.e., Pfizer-BioNTech or Moderna, are the preferred first line of COVID-19 vaccines as they have been studied extensively and have a wealth of data to support their usage [15]. In addition, Novavax is comparable to mRNA vaccines in terms of success, despite the fact that there is information on its safety and effectiveness is currently limited. The Janssen is appropriate for those without access to or with medical conditions that preclude the other three vaccines described above. Even though Janssen is the least preferred vaccine due to the potential for adverse events post-vaccination, it is still recommended to be vaccinated rather than foregoing COVID-19 vaccination altogether. More details on the Janssen vaccine are mentioned below under the section on contraindications, precautions and safety concerns. 

Studies have also shown that two doses of mRNA vaccine are more effective than a single dose of Janssen [17]. For instance, Pfizer-BioNTech and Moderna have 93% and 88% effectiveness respectively after two doses in lowering COVID-19-related hospitalizations and mortality. Comparatively, Janssen is only 71% effective in lowering hospitalizations and mortality linked to COVID-19 [17,18]. Observational studies performed to compare the effectiveness of the two mRNA vaccines show no clinically meaningful difference between the two mRNA vaccines [19].

COVID-19 vaccines: doses and intervals, monovalent and bivalent booster doses

**Figure 1:**
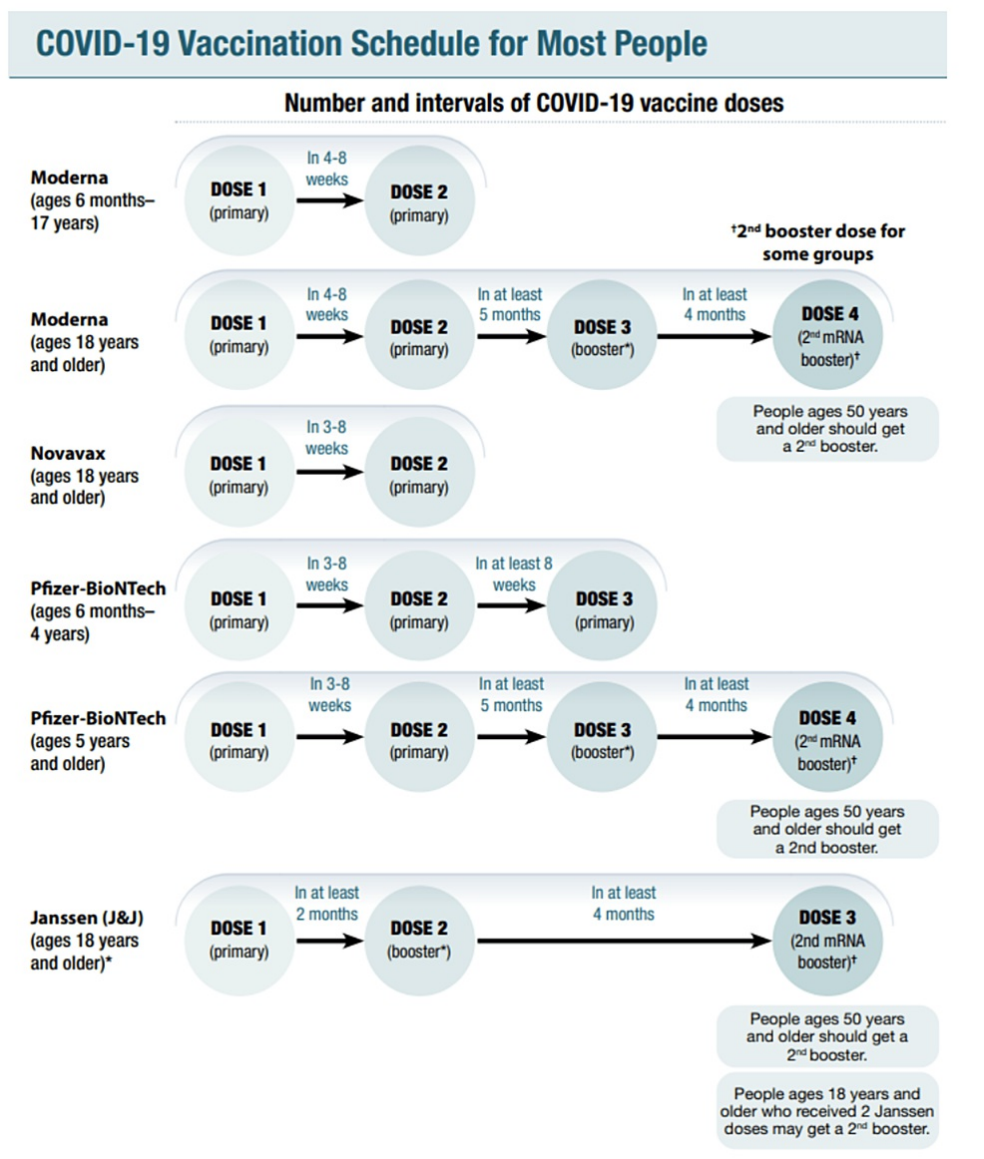
This image highlights the old COVID-19 vaccine schedule for immunocompetent individuals released by the CDC. [15]

**Figure 2:**
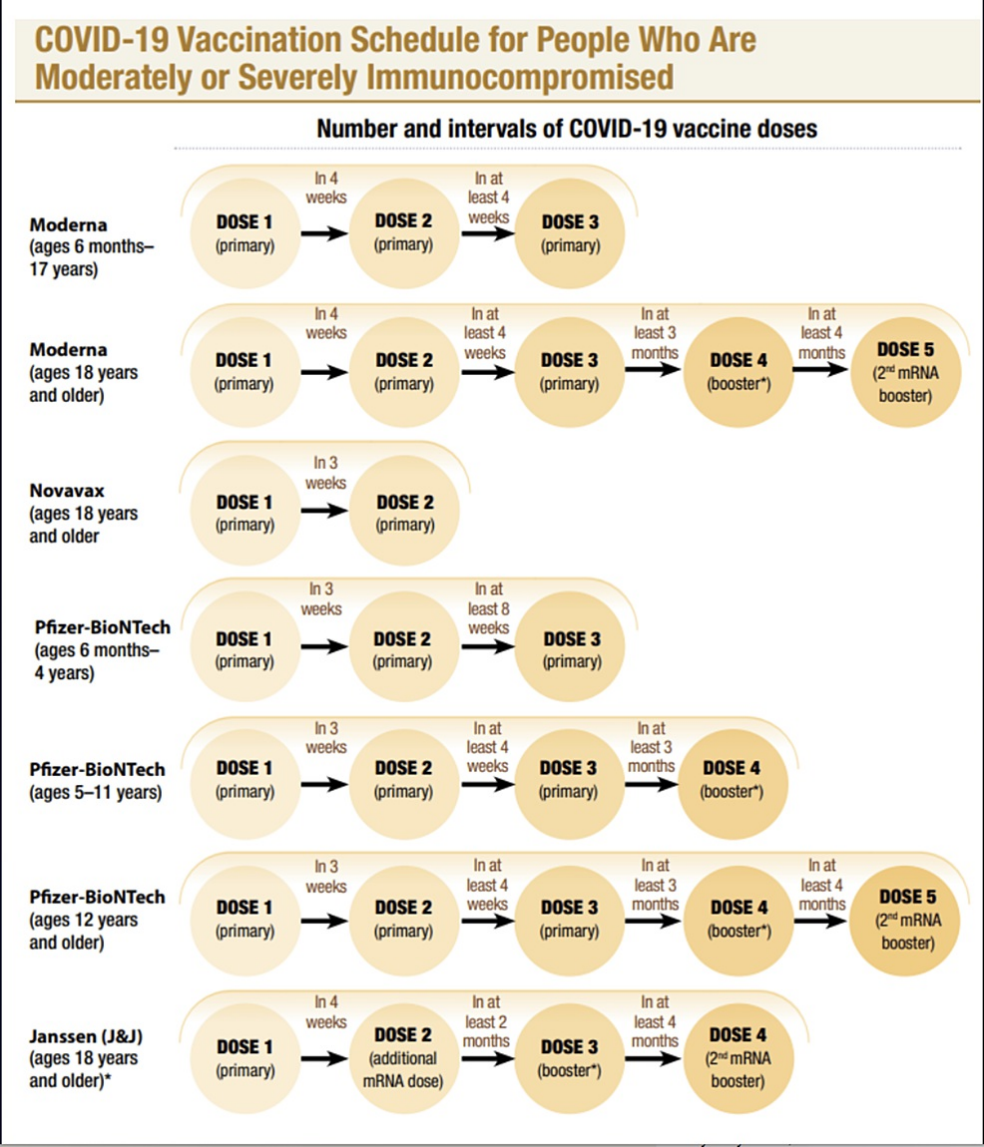
This image highlights the old COVID-19 vaccine schedule for immunocompromised individuals released by the CDC. [20]

The following is the description of the old COVID-19 immunization schedule approved by the CDC before August 31, 2022. This has been discussed below in detail and can also be seen in Figure 1 and Figure 2. 

The guidelines for the COVID-19 vaccines have not yet been granted FDA approval or authorization for use in children under six months. These guidelines are frequently updated by the CDC.

Pfizer-BioNTech COVID-19 (BNT162b2)

Pfizer-BioNTech COVID-19 (BNT162b2) vaccine is an mRNA-based vaccine that was developed by BioNTech and Pfizer in close collaboration [15]. This vaccine has been approved for ages six months and above [15,20]. The vaccine schedule is divided into four age groups, i.e., individuals 18 years or older, individuals aged 12 to 17 years, children aged five to 11 years, and children aged six months to four years. The duration of the primary series and the number of boosters doses required for each age group are different and therefore have been discussed below in detail.

Adults 18 years and older: In a healthy individual, the first two doses of the Pfizer-BioNTech vaccine are considered primary series, which can be given three to eight weeks apart. The individual is considered fully vaccinated two weeks after receiving the final dose in the primary series [15]. An immunocompromised individual will receive three doses of the primary series three to four weeks apart from each other [20]. A healthy individual's first booster dose is given at least five months after the last dose. The immunocompetent individuals will only qualify for a second booster vaccine if they are 50 years and older and at least four months after the first booster vaccine. However, in moderate to severely immunocompromised individuals, the first booster dose must be given at least three months after the second dose. The immunocompromised individual will receive their second booster dose four months after the first booster [15,20]. 

Children and teenagers age 12 to 17 years: In a healthy individual, the first two doses of Pfizer-BioNTech are considered primary series, which can be given three to eight weeks apart. The individual is considered fully vaccinated two weeks after receiving the final dose in the primary series [15]. However, in a moderate to severely immunocompromised individual, three doses of the primary series need three-week intervals between each one. In a healthy individual, only one booster dose is given at least five months from the last vaccine dose. However, in an immunocompromised patient, the first booster dose is still considered a primary dose and needs to be given within four weeks of the last vaccine [20]. The immunocompromised individual will receive their first booster in three months and the second in at least four months. 

Children age five to 11 years: In a healthy individual, the first two doses of Pfizer-BioNTech are considered primary series, which can be given three to eight weeks apart. The individual is considered fully vaccinated two weeks after receiving the final dose in the primary series. However, in an immunocompromised individual, three doses of the primary series need to be given three to four weeks apart [15]. In a healthy individual, only one booster dose is given at least five months from the last dose. However, in an immunocompromised patient, the booster dose must be given three months from the last dose [20]. 

Children age six months to four years: In healthy children, a total of three primary doses are given. The first two doses of Pfizer-BioNTech can be given three weeks apart, and the third can be given eight weeks from the last dose. The individual is considered fully vaccinated two weeks after receiving the final dose in the primary series. The immunocompromised individuals also receive the same three doses with the same duration in the primary series as healthy individuals [15]. No booster vaccine is recommended at this age range for both immunocompetent and immunocompromised individuals [20].

Moderna COVID-19 Vaccine (mRNA-1273)

Moderna COVID-19 vaccine (mRNA-1273) is also an mRNA-based vaccine developed by the company Moderna [15]. This vaccine, similar to Pfizer-BioNTech, has been approved for ages six months and above [15,20]. This vaccine is divided into two age groups only, i.e., individuals who are 18 years or older and individuals from age six months to 17 years. The duration of the primary series and the number of boosters doses required for each age group are different and therefore have been discussed below in detail [20].

Adults 18 years and older: In a healthy individual, for the age group 18 to 49 years, the primary series consists of two doses, and for individuals aged 50 years and older, three doses are given at least four to eight weeks apart. The individual is considered fully vaccinated two weeks after receiving the final dose in the primary series. However, in an immunocompromised individual, the three doses of the primary series need to be given at four-week intervals [15]. One booster dosage is administered to healthy adults between 18 to 49 years of age; those 50 and older receive two booster doses at intervals of four to five months from the previous dose [20]. However, in an immunocompromised patient, two booster doses should be administered at least three to four months apart from the last dose. 

Children and adolescents age six months to 17 years: In these healthy individuals, two doses of Moderna vaccine are considered primary series, should be given four to eight weeks apart. The individual is considered fully vaccinated two weeks after receiving the final dose in the primary series [15]. However, in an immunocompromised individual, three doses are given at four-week intervals to complete the primary series [20]. No booster vaccine is recommended for both immunocompetent and immunocompromised individuals under this age group.

Novavax COVID-19 Vaccine (NVX-CoV2373)

Novavax COVID-19 vaccine (NVX-CoV2373) is an adjuvanted recombinant protein vaccine developed by the company Novavax [15]. This vaccine has been approved for individuals 18 years and older only. The healthy individual will receive two doses of Novavax vaccine as primary series, given three to eight weeks apart [15]. The individual is considered fully vaccinated two weeks after receiving the final dose in the primary series [20]. However, in an immunocompromised individual, the two doses of the primary series must be given within three weeks. No booster vaccine is recommended for both immunocompetent and immunocompromised individuals.

Janssen COVID-19 Vaccine (Ad26.COV2.S)

Janssen COVID-19 vaccine (Ad26.COV2.S) is an adenoviral vector vaccine [15] that, like Novavax vaccine, is only approved for individuals 18 years and older. The healthy individual will receive only one dose of Ad26.COV2.S as primary series. However, in an immunocompromised individual, the person requires a second dose of an mRNA vaccine. After receiving the second dose of the mRNA vaccine, the immunocompromised individual is considered fully vaccinated [15]. A booster dose is not recommended in a healthy individual. However, in an immunocompromised individual, the first booster dose of Ad26.COV2.S is required at least two months after receiving the second dose in the primary series [20]. After that, a second booster dose of mRNA vaccine is again required at least four months after receiving the first booster dose.

New COVID-19 vaccine schedule with bivalent mRNA vaccine recommendations

**Figure 3:**
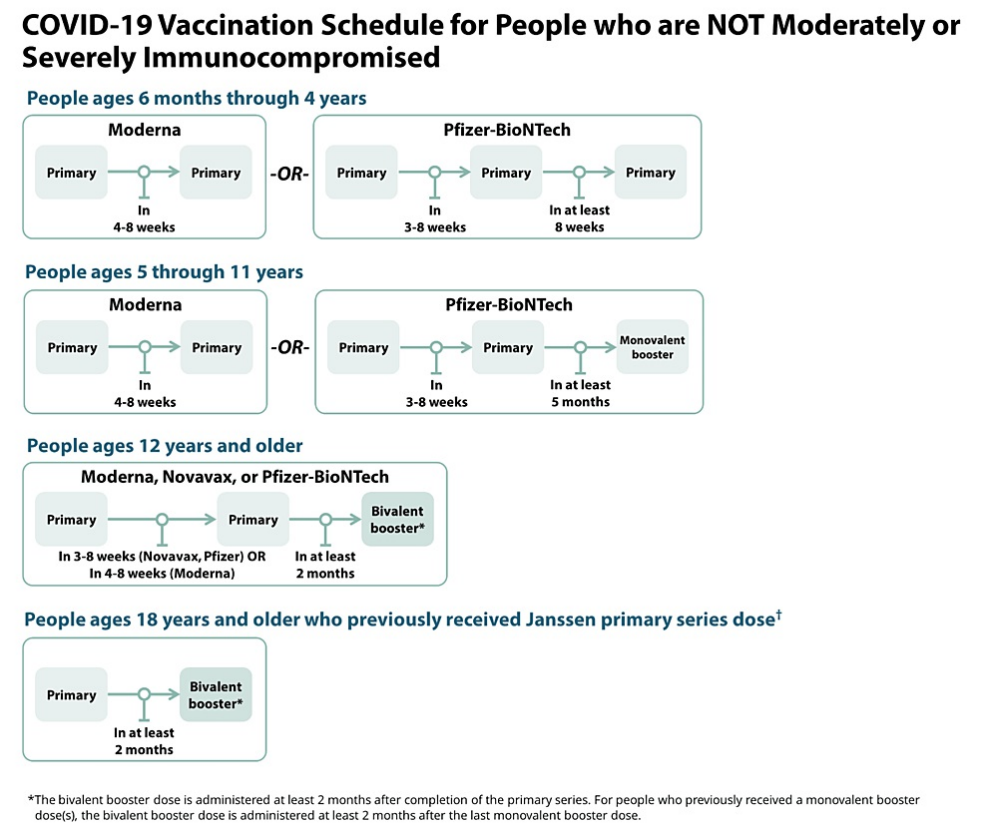
This image highlights the new COVID-19 vaccine schedule with bivalent booster dose for immunocompetent individuals released by the CDC. [15]

**Figure 4:**
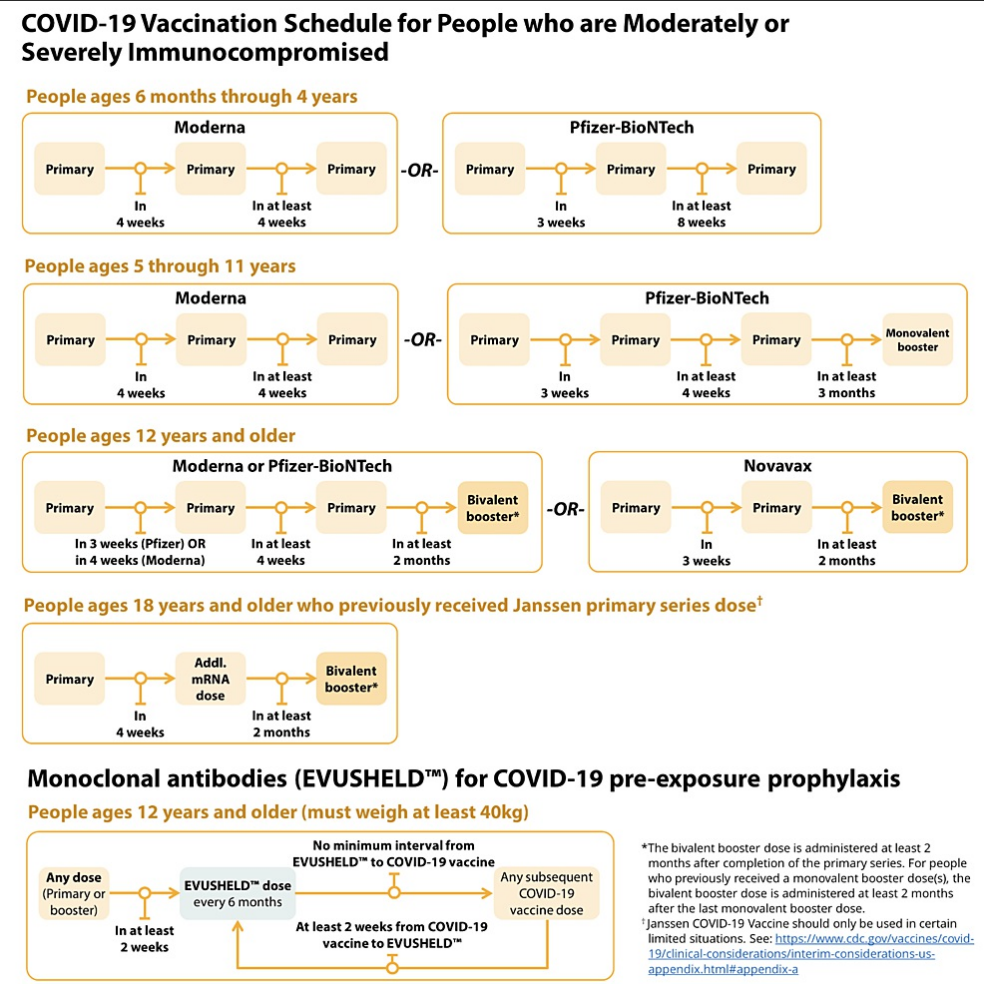
This image highlights the new COVID-19 vaccine schedule with bivalent booster dose for immunocompromised individuals released by the CDC. [20]

As of August 31, 2022, the FDA has authorized and approved the bivalent COVID-19 vaccine booster dose. The CDC has also updated its COVID-19 vaccine schedule and guidelines, especially focusing on booster doses, as seen in Figure 3 and Figure 4. The bivalent mRNA COVID-19 vaccine booster contains the original SARS-CoV-2 strain used in monovalent vaccines plus the newer variants of SAR-CoV-2, omicron BA.4, and BA.5. The benefit of receiving the new updated bivalent vaccine is to increase vaccine effectiveness against new variants of SAR-CoV-2 as well as improving the body's immune response to new emerging variants [15,20].

This recommendation extends to both healthy and moderately or severely immunocompromised individuals over the age of 12. The two bivalent mRNA vaccines currently available are Pfizer and Moderna. To determine which bivalent vaccine is most appropriate, healthcare providers should refer to the individual's age group and primary vaccine series type. 

The current recommendations are as follows:

In healthy, moderately, or severely immunocompromised individuals, the primary vaccination series has remained the same in all individuals (in the same age group). The only change is with the booster dose only. Instead of receiving multiple booster doses, CDC now recommends individuals ages 12 years and above receive only "one bivalent mRNA booster dose" after completing the primary series or any monovalent booster doses received in the past [15,20]. 

This new recommendation of receiving one bivalent booster dose replaces all prior monovalent mRNA booster vaccination for individuals ages 12 and above. The monovalent mRNA vaccine is not authorized as a booster dose for individuals ages 12 years and older.

Individuals ages five through 11 who received the Pfizer-BioNTech vaccine will still receive only the monovalent booster after completing the primary dose (not the bivalent). Also, all individuals who have received Novavax as a primary vaccine should receive a bivalent booster dose of either Pfizer or Moderna vaccine [15,20].

Can we mix different vaccines along with booster dose?

Unless otherwise noted, the CDC advises that the primary series of mRNA vaccinations be completed with the same type. For example, if a particular mRNA vaccine is in short supply or unavailable, CDC recommends delaying the second dose until the same vaccine can be obtained [21]. However, if someone receives the first dose of the mRNA vaccination but is unable to receive the second dose due to an allergic reaction or other medical issues, they are still permitted to receive Janssen 28 days after receiving the last dose of the mRNA vaccination, provided they are not also allergic to Janssen [21]. 

Per CDC recommendations, an alternative vaccine to the one given as part of the primary series can be administered as a booster. This is particularly relevant when an mRNA vaccination is administered as a booster to people who had Janssen as their primary series. Studies have demonstrated that this strengthens vaccine effectiveness against COVID-19 viral infection [21]. The interval for the booster dose should remain the same, as discussed above. Nonetheless, there is no significant difference when the alternate mRNA vaccine is mixed in individuals who have received either the primary Pfizer-BioNTech or Moderna vaccine.

Can you administer non-COVID-19 vaccines with the COVID-19 vaccines?

Per CDC guidelines, non-COVID-19 vaccines can be administered simultaneously with the COVID-19 vaccine. However, both vaccines should be injected at least in different limbs (if they are associated with local reactions) or at least 1 inch apart [21].

Adverse reactions, contraindications, and precautions

The safety of COVID-19 vaccinations has been the subject of numerous misconceptions and myths, but in reality, most of them are relatively safe. Table 2 and Table 3 summarize contraindications and precautions associated with different COVID-19 vaccines in further detail. 

**Table 2:** Contraindications and precautions associated with different COVID-19 vaccine types and their associated recommendations for future vaccination. [21,22]

Vaccines	Pfizer, (mRNA)	Moderna (mRNA)	Janssen	Novavax	Recommendation
Contraindications					
Prior episode of a severe allergic reaction (anaphylaxis) from an earlier COVID-19 vaccine.	X	X	X	X	Patients should not receive any more doses of that same vaccine type. If an allergic reaction is experienced after an mRNA vaccine, the Janssen vaccine may be used for subsequent doses. Please consult an allergist for further testing.
Prior history of allergy to an ingredient in the COVID-19 vaccine	X	X	X	X	Patients should not receive any additional doses of that same vaccine type. Please consult an allergist for further testing.
Thrombosis with thrombocytopenia (TTS) development after COVID-19 vaccination			X		If TTP occurs, future Janssen doses are contraindicated. To complete vaccination series or receive booster doses, mRNA vaccines are recommended after recovery.
Guillain-Barre Syndrome (GBS) development after COVID-19 vaccination			X		Future doses of Janssen vaccines are not recommended. These patients should receive an mRNA vaccine to complete their vaccination series or as boosters.
Previous experience of immune-mediated thrombosis & thrombocytopenia (ex. HIT)			X		Future doses of Janssen vaccines are not recommended. These patients should receive an mRNA vaccine to complete their vaccination series or as boosters.
Precautions					
Prior episode of allergic reaction to prior COVID or non-COVID 19 vaccine and/or other injected treatments	X	X	X	X	For people who have had a severe allergic reaction to a COVID-19 vaccine, precautions should be taken to determine which other COVID-19 vaccine type would be most appropriate for further doses.
Prior episode of Guillain-Barre Syndrome (GBS) not related to the COVID-19 vaccine			X		mRNA vaccines are preferred in patients with a history of GBS. Administration of the Janssen vaccine should be approached with caution.
Myocarditis/Pericarditis after COVID-19 vaccination in adolescents, and young males	X	X		X	If myocarditis or pericarditis occurs, future doses of any mRNA or Novavax COVID-19 vaccines are not recommended. Janssen is recommended for later doses and boosters after recovery.

**Table 3:** Common side effects of the COVID-19 vaccines and their management. [21-27]

Vaccine	Common Side Effects by Age Group	Treatment of Common Side Effects
All Vaccine types (Moderna, Pfizer, Janssen, Novavax)	*For Age: 6 months to 3 years:* Pain at the site of injection, lymph node swelling, fatigue, decreased appetite, irritability. *For Age: 4 to 17 years:* Pain at the site of injection, fatigue, migraines/headache, chills, lymph node swelling, muscle/joint pain. *For Age: Adults 18+ years:* Pain at the site of injection, migraines/headache, chills, elevated temperature/fever, fatigue, nausea, muscle/joint pain	For pain at the injection site, use a wet and cool cloth at the site and move your arm. For fever, stay hydrated with fluids. Medications (e.g., Ibuprofen, acetaminophen, antihistamines) can be used after vaccination but not to prevent post-vaccination symptoms. If pain or redness at the injection site worsens after one day or if symptoms do not improve after a few days, please consult a healthcare provider.
Vaccine	Rare Side effects	Treatment of rare side effects
All Vaccine types (Moderna, Pfizer, Janssen, Novavax)	Anaphylaxis: Respiratory - trouble breathing, wheezing, stridor, difficulty swallowing Cardiovascular - decreased blood pressure, fainting Skin - hives, itching, mouth or face swelling.	Please seek immediate medical attention. Contact emergency medical services (EMS) and place patients in an upward-facing position. Assess airway, breathing, circulation, and mental status. Administer epinephrine (1:1000 dilution) via IM injection immediately. Administer every 5-15 minutes if symptoms do not improve while waiting for EMS. Avoid any further vaccination. Follow up with an allergist.
Pfizer/Moderna/Novavax	Myocarditis/Pericarditis: Chest pain Trouble breathing Pounding heart/Palpitations.	Please seek immediate medical attention. Obtain an ECG, troponin status, and markers of inflammation (ESR, CRP); Consider Cardiology evaluation.
J&J	*Thrombosis with thrombocytopenia syndrome (TTS): *Headache, trouble with vision, abdominal pain, trouble breathing, recent bruising/bleeding, leg or back pain. *Guillain-Barre Syndrome (GBS): *trouble with walking or facial movements, issues with controlling the bladder or bowels, decreased strength or tingling in the arms or legs.	*TTS:* Please seek immediate medical attention. Consult hematology and obtain a CBC, platelet count, and imaging to confirm thrombosis. *GBS: *Please seek immediate medical attention. Begin treatment (either plasma exchange or high-dose immunoglobulin therapy (IVIg)) as soon as possible.

Severe allergic reaction to COVID-19 vaccine: signs, symptoms, management, patient counseling and precautions to take

A history of severe allergic responses, including anaphylaxis, to a previous COVID-19 vaccine or a vaccine ingredient is an important contraindication to future COVID-19 vaccine doses [21]. For example, adjuvants used in COVID-19 vaccines to boost the desired immune response can occasionally induce an allergic or autoimmune reaction in specific individuals. These include polysorbate in Novavax and Janssen and polyethylene glycol (PEG) in Pfizer-BioNTech and Moderna [22,26].

Anaphylaxis has an acute onset from minutes to several hours and requires immediate intervention [24,21]. Symptoms include respiratory involvement (trouble breathing, wheezing, stridor, difficulty swallowing), cardiovascular involvement (decreased blood pressure, fainting, dizziness), and skin involvement (hives, itching, mouth or face swelling) [21,24]. If anaphylaxis occurs post-vaccination, it is typically 15 to 30 minutes after the vaccine dose and warrants immediate management [21]. Management includes assessing airway, breathing, circulation, change in mental status, contacting emergency medical services (EMS), placing the patient in an upward-facing position, and immediately administering epinephrine (1:1000 dilution) via IM injection [21]. Symptoms typically improve after the first dose of epinephrine; if not, a repeat dose may be administered every five to 15 minutes while waiting for EMS to arrive [21]. Although anaphylaxis is a potentially life-threatening condition, it is a rare adverse effect with an estimated incidence of eight cases per million [27].

Per CDC, if a patient undergoes a severe allergic reaction after receiving a COVID-19 vaccine, they should not have any more doses of that same vaccine type and should be referred to an allergist for additional follow-up [21]. For example, if an allergic reaction is experienced after receiving an mRNA vaccine, the patient should not have any further doses of any type of mRNA COVID-19 vaccine [21]. After receiving the first dose of the Pfizer-BioNTech or Moderna vaccine, individuals who experience an immediate allergic reaction may be able to receive a dose of Janssen to complete their vaccination series [22]. 

In a multisite US study conducted from January 2021 to March 2021, the individuals who received the first dose of mRNA vaccine and experienced severe allergic reactions were able to successfully receive a second dose of Pfizer-BioNTech or Moderna vaccines [28]. This study suggests that the reactions seen after the initial dose are not true IgE-mediated allergic reactions and are likely non-IgE mediated [28]. 

A general precaution prior to receiving a COVID-19 vaccine is evaluating for a history of an allergic reaction to a past COVID-19 or non-COVID-19 vaccine and other injected treatments [21]. For individuals with a history of severe allergic reactions to a COVID-19 vaccine, precautions should be taken to determine which COVID-19 vaccine type would be most appropriate for further doses [21]. For example, a polysorbate allergy is a precaution for Pfizer-BioNTech or Moderna because they contain the closely related protein PEG [21]. If an individual experiences an immediate allergic reaction to a non-COVID-19 vaccine or other injectable therapy due to either an unknown or known component of a COVID-19 vaccine, it may help to follow up with an allergist-immunologist for further testing [21].

Thrombosis with thrombocytopenia syndrome (TTS) from COVID-19 vaccine

TTS is also known as vaccine-induced thrombotic thrombocytopenia (VITT). Studies show that the Janssen vaccination led to an estimated four cases per million and 0.6 deaths per million after the initial dose [29]. 

Signs, Symptoms, and Management of TTS

Symptoms of TTS usually develop between seven to 14 days after vaccination and include headache, vision changes, abdominal pain, shortness of breath, trouble with bleeding or bruising, and pain in the legs or back [29,30]. If there is suspicion of VITT, blood work such as a CBC and platelet count must be obtained immediately, and imaging to identify thrombosis based on patient presentation [30]. If VITT is present, immediate consultation with a hematologist is warranted [30].

Patient Counseling After TTS Occurs

If TTS is experienced after receiving a Janssen vaccine, a subsequent dose with it is contraindicated [21]. Those who wish to complete their vaccination series should receive an mRNA booster dose at least two months after the first dose of Janssen and only if they have recovered [21]. Ultimately, the patient should discuss with their hematologist and healthcare providers to decide which mRNA vaccine is most appropriate and the optimum interval of booster administration [21]. Although the exact mechanism behind the development of TTS is unclear, it is hypothesized to be similar to heparin-induced thrombocytopenia (HIT) due to the presence of platelet factor 4 antibodies in cases of TTS [21,30]. This explains why patients should not get another dose of Janssen who have experienced a case of immune-mediated thrombosis and thrombocytopenia, such as HIT [21]. This does not include a history of other blood clotting conditions such as thrombophilia, pregnancy, or the use of birth control since they do not have an increased risk of developing TTS [21]. 

Guillain-Barre syndrome (GBS) from COVID-19 vaccine

If an individual develops GBS within six weeks of administration of a Janssen vaccine, it is not recommended to administer another dose of it [21]. By July 2021, the Vaccine Adverse Event Reporting System (VAERS) approximated one Guillain Barre Syndrome (GBS) case per 100,000 doses [31]. 

Signs, Symptoms, and Management of GBS

GBS is a peripheral disorder involving nerve damage caused by one’s immune system [21]. Symptoms typically occur within 42 days after a dose of Janssen, with males from ages 40-64 years most affected [21]. This can lead to walking difficulties, facial weakness, bladder or bowel dysfunction, bilateral upper and lower extremity weakness, and tingling sensations. [21]. The presence of any of these symptoms following the Janssen vaccine warrants immediate medical attention [21]. Treatment is most effective when initiated within two weeks of symptom onset and involves either plasma exchange or high-dose immunoglobulin therapy (IVIG) [32].

Patient Counseling After GBS Occurs

For those that develop GBS within six weeks after Janssen dose administration and wish to complete their vaccination series, an mRNA COVID-19 vaccine should be used for booster doses [21]. For future doses, mRNA vaccines are also recommended for those that develop GBS after six weeks of receiving a Janssen dose [21]. 

GBS Precautions

For individuals with a history of GBS, the administration of Janssen should be approached with caution [21]. Since using mRNA vaccines does not increase the probability of contracting GBS, Pfizer-BioNTech, Moderna, or Novavax are recommended in these situations [21].

Myocarditis/pericarditis from COVID-19 vaccine

mRNA COVID-19 vaccines and Novavax have been associated with developing myocarditis or pericarditis in young males 12 to 39 years of age post-vaccination [21,33]. However, to put it in perspective, COVID-19 infection has a greater likelihood of causing such a cardiac adverse event than vaccination. Reports of myocarditis to VAERS between December 2020 and August 2021 showed that among males, cases ranged from 52-106 per million doses of mRNA vaccines between the ages of 12 to 24 [34]. An eight-week dosage interval is now suggested between the first and second dose of mRNA or Novavax to reduce the risk of vaccine-related myocarditis for individuals aged six months to 64 years and especially for males 12 to 39 years old [21].

Signs, Symptoms, and Management of Myocarditis

Myocarditis occurs when cardiac muscle is inflamed, while pericarditis occurs when the sac around the heart is inflamed [33]. Individuals, especially young men who have received a recent COVID-19 vaccine and who are experiencing chest pain, trouble breathing, and a pounding heart or palpitations, should seek medical attention [33]. Management should consist of cardiology consultation and a cardiac work-up, an EKG, and blood tests, which include troponin level and inflammatory markers such as erythrocyte sedimentation rate (ESR) vs. C-reactive protein (CRP) [33-36]. 

Patient Counseling After Myocarditis Occurs

If a patient experiences myocarditis, it is advised that subsequent doses of any mRNA or Novavax COVID-19 vaccines be avoided [21]. If an individual that has developed myocarditis would like to receive another COVID-19 vaccine, they need to fully recover and be stable [21]. For subsequent doses or boosters, Janssen is recommended over mRNA or Novavax vaccines [21]. Individuals with a history of myocarditis or pericarditis unrelated to COVID-19 vaccination can get any COVID-19 vaccine that is FDA-approved after they have recovered from their condition [21].

Other widely used COVID-19 vaccines worldwide

There are seven other COVID-19 vaccines available worldwide which are listed in Table 4.

**Table 4:** Other types of COVID-19 vaccine available worldwide. [37-45]

Brand Name	Type	Key Feature
ChAdOx1 nCoV-19/AZD1222 (AstraZeneca)	Non-Replicating Viral Vector based, utilizing adenovirus as vector	2 dose vaccine given 8-12 weeks apart Refrigerator storage at (2 to 8 °C)
Convidecia Or AD5-nCOV (CanSino Biologics)	Non-Replicating Viral Vector based, utilizing Adenovirus 5 vector	Single-dose vaccine Refrigerator storage at (2 to 8 °C)
Gam-COVID-Vac/Sputnik V (Gamaleya Institute)	Non-Replicating Viral Vector based, utilizing adenovirus - Ad26 and Ad5	2 dose vaccine given 21 days to 3 months apart Refrigerator storage at (2 to 8 °C)
WIV04 and HB02 (Sinopharm)	inactivated virus particle vaccine based on two different COVID-19 isolates	2 dose vaccine given 28 days apart Refrigerator storage at (2 to 8 °C)
CoronaVac (Sinovac)	Inactivated virus with an Aluminum hydroxide as an adjuvant	2 dose vaccine given 28 days apart Refrigerator storage at (2 to 8 °C)
Covaxin	inactivated virus particle vaccine	2 dose vaccine given 29 days apart Refrigerator storage at (2 to 8 °C)
ZyCoV-D (Zydus Cadila)	plasmid-based DNA vaccine	3-dose vaccine with each dose given 28 days apart Uses needleless injector, therefore, painless Refrigerator Storage at (2 to 8 °C)

ChAdOx1 nCoV-19/AZD1222 (AstraZeneca - the University of Oxford and the Serum Institute of India)

The University of Oxford and AstraZeneca collaborated to create this vaccine. This vaccine utilizes DNA to create S-proteins as opposed to mRNA vaccines. The ChAdOx1 chimpanzee adenovirus serves as the vector for this vaccine. The genetic code for the COVID-19 S-protein will then be injected by the modified adenovirus into the nucleus of the recipient's cells, allowing it to be translated into mRNA. Later, this mRNA is translated into spike proteins that travel to the surface, where the immune system can detect them and eventually mount an immunological response. It is simple to use and store this vaccine. The vaccine does not have to remain frozen and can last long, even when refrigerated at 2°C to 8°C. The WHO recommends this vaccine be given in two doses IM eight to 12 weeks apart [35-37]

Ad5-Based COVID-19 Vaccine (CanSino Biologics)

This vaccine was developed by CanSinoBio and the Beijing Institute of Biotechnology as a single-dose vaccine against COVID-19 in China. This single-dose vaccine is given IM and is based on the replication of an adenovirus type 5 vector that expresses the S-protein of SARS-CoV-2. This vaccine is available in China and other countries, including Pakistan and Mexico [38]. The efficacy of this vaccine showed 57.5% and 91.7% efficacy, respectively, in preventing symptomatic and severe diseases during the phase 3 trial. Further study must be performed to check the effectiveness against different COVID-19 variants [38].

Gam-COVID-Vac/Sputnik V (Gamaleya Institute)

This vaccine was developed by the Gamaleya Research Institute of Russia. The vaccine's mechanism of action is similar to AZD1222 discussed above. Sputnik V uses double-stranded DNA for building COVID-19 spike proteins to induce an immune response. The vector used for this vaccine is also Adenovirus 26 (AD26) and Adenovirus 5 (AD5). This vaccine is given IM in two doses. The initial dose contains the AD26 vector followed by the AD5 vector given 21 days to three months apart [39,40]. The vaccine showed 91.6% efficacy in preventing symptoms of COVID-19 after the second dose. Further study needs to be performed to check the effectiveness against different COVID-19 variants [39,40].

WIV04 and HB02 (Sinopharm)

The Beijing Institute of Biological Products of Sinopharm was tasked with developing these two inactivated virus particle vaccines based on two different COVID-19 isolates found in patients in China. These two vaccines are given IM 28 days apart [41]. The vaccine showed 73% effectiveness for WIV04 and 78% effectiveness for HB02. Further study must be performed to check the efficacy against different COVID-19 variants [41]. This vaccine is available in China and other countries like the United Arab Emirates.

CoronaVac (Sinovac)

This vaccine is based on an inactive COVID-19 virus within an aluminum hydroxide as an adjuvant. This is a two-dose vaccine given IM 28 days apart [42]. This vaccine showed 83.5% efficacy in a phase 3 trial. However, other countries like Brazil and Chile have reported lower vaccine efficacy use, around 70% [42,43].

Covaxin (BBV152: Bharat Biotech & Indian Council of Medical Research)

This vaccine is based on an inactive COVID-19 virus developed by Bharat biotech and the Indian Council of Medical Research. This is a two-dose vaccine given IM 29 days apart [44]. The vaccine showed 78% efficacy in a randomized trial [44]. Further study must be performed to check the effectiveness against different COVID-19 variants.

ZyCoV-D (Zydus Cadila)

This vaccine is considered the world's first plasmid-based DNA vaccine developed by Zydus Cadila in India against the COVID-19 virus. This vaccine uses a plasmid that carries a coronavirus genome to generate S-proteins in the host cell, leading to an immune response. It is a three-dose vaccine with each dose given 28 days apart. The vaccine uses a needleless injector that delivers the vaccine with a high-pressure stream subcutaneously. The benefit of this vaccine is that it is needleless and, therefore, painless. Also, since this vaccine is plasmid-based, vector-based immunity concerns can be avoided [45]. This vaccine has shown to be 67% effective against COVID-19 infection. Further study must be performed to check the effectiveness against different COVID variants [46].

COVID-19 vaccine effectiveness

In the section, we have discussed the effectiveness of different COVID-19 vaccines against different SARS-CoV-2 variants [46-49]. This does not include studies from bivalent COVID-19 booster doses.

Delta (B.1.617.2) Variant

Several SARS-CoV-2 variants have surfaced since the COVID-19 pandemic erupted, despite vaccine efforts to confine it. Critical S-protein mutations (D614G, L452R, P681R, and T478K) have been linked to the delta variant's increased transmissibility. Another major factor in the reduced vaccine efficacy against delta variants has been theorized to be its increased affinity of the S-protein and ACE2. The table highlights the vaccine effectiveness against the delta variant. The mRNA vaccines were over 94% effective two to four weeks after completing two doses. The effectiveness goes down to around 80% at 25 weeks or more after the completion of two doses of mRNA vaccines. The vaccine effectiveness increases to over 95% when boosted with either of the mRNA vaccines.

After two doses, the AstraZeneca vaccine showed 82% effectiveness against the delta variant. This effectiveness goes down to around 43.5% at 25 weeks or more. The vaccine effectiveness increases to over 95% when boosted with either of the messenger RNA vaccines as shown in Table 5.

**Table 5:** Vaccine effectiveness after two doses of vaccine followed by a booster dose of either Pfizer or Moderna against the delta variant. [46-49]

Vaccine Type	2 doses of vaccine		Booster dose	
	2-4 weeks after 2nd dose	25 weeks after 2nd dose	Pfizer (5-9 weeks after booster dose)	Moderna (5-9 weeks after booster dose)
Pfizer-BioNTech	>94%	~80%	>95%	>95%
Moderna	>94%	~80%	>95%	>95%
AstraZenac	~82%	~43.5%	>95%	>95%

Omicron (B.1.1.529) Variant

According to CDC data from August 2022, BA.5 sub-lineages of the omicron variety accounted for around 88% of infections in the United States. The effectiveness of vaccines was lower for the omicron variant for all mRNA vaccines compared to the delta variant. Patients who received two doses of Pfizer-BioNTech vaccine initially had vaccine effectiveness of 65.5% around two to four weeks after completing the second dose. This effectiveness goes down to 8.8% after 25 weeks. Similarly, with the Moderna vaccine, the two doses of vaccine were initially effective at 75.1% two to four weeks after completing the second dose. This effectiveness reduces to 14.9% after 25 weeks as shown in Table 6. To combat these mutations, vaccine manufacturers are constantly developing next-generation boost doses.

**Table 6:** Vaccine effectiveness after two doses of either Pfizer or Moderna against the omicron variant. [46-49]

Vaccine Type	2-4 weeks after 2nd dose	25 weeks after 2nd dose
Pfizer-BioNTech	~65.5%	~8.8%
Moderna	~75.1%	~14.9%

For the individuals who received Pfizer-BioNTech as a primary series (two doses) and as a booster dose, the vaccine effectiveness increased to around 67% two to four weeks post booster dose. This effectiveness declined to about 45% after 10 weeks of receiving the booster dose as shown in Table 7.

**Table 7:** Vaccine effectiveness after two primary doses of Pfizer vaccine followed by a booster dose of Pfizer against the omicron variant. [46-49]

Vaccine Type	2-4 weeks after 2nd dose	25 weeks after 2nd dose	Pfizer Booster (2-4 weeks after booster dose)	Pfizer Booster (10 weeks after booster dose)
Pfizer	~65.5%	~8.8%	~67%	~45%

The individual who received Pfizer-BioNTech as a primary course (two-dose vaccine) and then received the Moderna vaccine as a third booster dose showed the effectiveness increased to around 74% two to four weeks after receiving the booster dose, which then declined to 64% around five to nine weeks as shown in Table 8.

**Table 8:** Vaccine effectiveness after two primary doses of Pfizer vaccine followed by a booster dose of Moderna against the omicron variant. [46-49]

Vaccine Type	2-4 weeks after 2nd dose	25 weeks after 2nd dose	Moderna Booster (2-4 weeks after booster dose)	Moderna Booster (5-9 weeks after booster dose)
Pfizer	~65.5%	~8.8%	~74%	~64%

The individuals who received Moderna as a primary course (two-dose vaccine) and then received Moderna vaccine as a third booster dose showed effectiveness increased to around 66% two to four weeks after receiving the booster dose as shown in Table 9. No further studies were done to see the effectiveness after four weeks.

**Table 9:** Vaccine effectiveness after two primary doses of Moderna vaccine followed by a booster dose of Moderna against the omicron variant. [46-49]

Vaccine Type	2-4 weeks after 2nd dose	25 weeks after 2nd dose	Moderna Booster (2-4 weeks after booster dose)
Moderna	~75.1%	~14.9%	~66%

The individuals who received Moderna as a primary series (two doses) and then received Pfizer-BioNTech vaccine as a third booster dose showed the effectiveness increase to around 65% at two to four weeks post booster dose as shown in Table 10. No further studies were done to see the effectiveness after four weeks.

**Table 10:** Vaccine effectiveness after two primary doses of Moderna vaccine followed by a booster dose of Pfizer against the omicron variant. [46-49]

Vaccine Type	2-4 weeks after 2nd dose	25 weeks after 2nd dose	Pfizer Booster (2-4 weeks after booster dose)
Moderna	~75.1%	~14.9%	~65%

AstraZeneca vaccine failed to provide any protection against this variant after 20 weeks. However, for individuals who have received AstraZeneca as a primary series (two doses) and then received the Pfizer-BioNTech vaccine as a third booster dose, the vaccine effectiveness increased to around 62% at two to four weeks post booster dose. This effectiveness declined to around 40% at 10 weeks. On the other hand, individuals who have received the Moderna vaccine as a booster dose had the vaccine effectiveness increased to 70% at two to four weeks post booster dose. This effectiveness declined to around 61% at five to nine weeks as shown in Table 11 and Table 12.

**Table 11:** Vaccine effectiveness after two primary doses of AstraZeneca vaccine followed by a booster dose of Pfizer against the omicron variant. [46-49]

Vaccine Type	20 weeks after 2nd dose	Pfizer Booster (2-4 weeks after booster dose)	Pfizer Booster (10 weeks after booster dose
AstraZeneca	~0%	~62%	~40%

**Table 12:** Vaccine effectiveness after two primary doses of AstraZeneca vaccine followed by a booster dose of Moderna against the omicron variant. [46-49]

Vaccine Type	20 weeks after 2nd dose	Moderna Booster (2-4 weeks after booster dose)	Moderna Booster (5-9 weeks after booster dose)
AstraZeneca	~0%	~70%	~61%

Limitations of this review

We have reviewed current literature regarding COVID-19 vaccines that have already been approved in the US as well as other nations like the UK, China, and India; some other countries may also have approved them. Not all vaccines licensed elsewhere in the globe could be reviewed in this article. Vaccine side effects discussed are from currently reported data as of September 9th, 2022, which may change in the future as new data emerges. 

## Conclusions

The COVID-19 pandemic continues to perpetuate with the emergence of variants of concern like the delta and the omicron variants, making robust preventative measures for virus containment more crucial than ever. While these mutations may not necessarily make the COVID-19 virus more virulent, they certainly hinder pandemic containment measures. The essential primary prevention method is still vaccination, although this virus is constantly mutating, making it possible to escape vaccine protection and cause vaccine breakthrough infections. Immunizations among those who qualify can prevent disease transmission, hospitalizations, and fatalities from COVID-19. Vaccine manufacturers continue to update COVID-19 vaccines to combat the variants. Four vaccines are currently approved for use in the United States by the FDA, giving people a choice of which one is appropriate. While most adverse effects are mild and self-limiting, it is essential to inform vaccine recipients of any potential adverse effects.

Additionally, compared to COVID-19 infection, the incidence of several potential side effects, such as myocarditis, is lower with COVID-19 immunization, as discussed previously in the article. Further, completing recommended doses (primary and booster) at the recommended interval is crucial to get the most benefit from the vaccination. People who have received the recommended number of vaccine doses fare substantially better than those with partial immunizations in the long term. In conclusion, the advantages of COVID-19 vaccination greatly outweigh the risks of adverse reactions. With COVID-19 vaccination reaching all parts of the world through global collaboration, prompt recognition of the adverse effects and appropriate screening of the population is essential for improving its acceptability and will be critical to containing this pandemic.
